# Correction of Excessive Tibial Plateau Angle and Limb Shortening in a Juvenile Dog Using a Hinged Circular Fixator Construct and Distraction Osteogenesis

**DOI:** 10.1155/2019/1439237

**Published:** 2019-11-16

**Authors:** Daniel D. Lewis, Stanley E. Kim, Justin Shmalberg, Sandra L. MacArthur

**Affiliations:** ^1^Department of Small Animal Clinical Sciences, College of Veterinary Medicine, University of Florida, Gainesville, FL, USA; ^2^Department of Comparative Diagnostic and Population Medicine, College of Veterinary Medicine, University of Florida, Gainesville, FL, USA

## Abstract

An 18-week-old Rhodesian Ridgeback puppy that was hit by a car sustained a Salter-Harris type III fracture of the left proximal tibial physis and ipsilateral diaphyseal femoral and tibial fractures. The diaphyseal fractures were successfully stabilized with bone plate fixation. Premature closure of the caudal aspect of the proximal tibial physis, secondary to the proximal physeal fracture, resulted in an excessively high tibial plateau angle (TPA) of 50° with a limb length discrepancy of 13% by 24 weeks of age. The deformity was addressed by performing a proximal tibial osteotomy and subsequent distraction osteogenesis to reduce the TPA while concurrently lengthening the crus. A radial osteotomy was performed in the proximal metaphyseal region and the hinged fixator was applied. Distraction was initiated the day following surgery at a rate of 1 mm per day as measured along the caudal cortex of the tibia with a rhythm of three distractions daily. Distraction was terminated 19 days postoperatively. Sequential distraction of the osteotomy resulted in 17 mm of tibial lengthening and a final TPA of 3°. The fixator was removed 52 days after application. Complications included wire tract inflammation involving the wires securing the proximal segment and a calcaneal fracture which required bone plate stabilization. The left pelvic limb was only 8% shorter than the right pelvic limb and the dog had only a subtle lameness 12 months after surgery. The hinged circular fixator construct allowed for both the reduction of the TPA and limb segment lengthening in this dog.

## 1. Introduction

Premature closure of the caudal portion of a dog's proximal tibial physis can result in an excessively high tibial plateau angle (ehTPA) which may precipitate cranial cruciate ligament (CCL) insufficiency [1–4]. Premature closure of the proximal tibial physis at an early age may also induce substantial limb shortening. Several surgical procedures have been described to address an ehTPA, such as modifications of the tibial plateau leveling osteotomy (TPLO) including a secondary osteotomy, cranial closing wedge osteotomy, and center of rotation of angulation (CORA)-based leveling osteotomy [4–7]. All of these described techniques involve acute correction of an ehTPA, but do not address, and may actually contribute to, limb length discrepancies. This report describes the use of a hinged circular external fixator and distraction osteogenesis to address an ehTPA that was a sequela to premature closure of the caudal portion of the proximal tibial physis in a juvenile dog. This approach was elected to reduce the tibial plateau angle (TPA) while increasing crural length, as the affected limb was already shorter than the normal, contralateral pelvic limb.

## 2. Case Report

A 24-week-old, 12.4 kg, spayed female, Rhodesian Ridgeback was presented to the University of Florida Small Animal Orthopedic Service for evaluation of a left pelvic limb lameness. The dog had sustained comminuted diaphyseal fractures of the left femur, tibia, and fibula when hit by a car at 18 weeks of age. The femur and tibia were plated and the fractures obtained radiographic union 4 weeks after surgery. The dog also sustained a Salter-Harris type III fracture of the caudal aspect of the left proximal tibial epiphysis as well as a left distomedial talar fracture with a proximal intertarsal joint intra-articular fracture fragment. These latter two fractures were not addressed surgically.

The dog was evaluated 6 weeks after the fractures had been repaired and had a mild left pelvic limb lameness, characterized by reduced extension of the left hip and reduced flexion of the left stifle, which was evident at a trot. The dog placed more weight on the right pelvic limb than the left while standing with the left pelvic limb maintained in slight internal rotation and abduction. The owner reported that the dog's lameness became more obvious with unrestricted activity. There was 2–3 mm of cranial drawer in both stifles which had a distinct end point, consistent with an expected amount of craniocaudal laxity present in a skeletally immature dog [8]. Cranial tibial thrust was not elicited in either stifle. Neither stifle had medial buttress, but pain was consistently elicited on hyperextension of the left stifle. Force plate analysis was performed at a trot and the peak vertical force (expressed as % body weight) was 56.9 and 80.1 for the left and right pelvic limbs, respectively. The vertical impulse (expressed as % body weight × second) was 5.7 and 10.0 for the left and right pelvic limbs, respectively.

Radiographs obtained at the time of the initial evaluation revealed remodeling of the healed left diaphyseal femoral, tibial, and fibular fractures (Figure 1). The left femur (142.6 mm) was 31.0 mm shorter than the right femur (173.6 mm) which was partially ascribed to difficulty in restoring femoral length during stabilization of the comminuted fracture. The left tibia (163.3 mm) was 13.2 mm shorter than the right tibia (176.5 mm) which was primarily ascribed to premature closure of the proximal tibial physis. There was 8° of varus angulation and slight internal rotation of the distal tibia. The left TPA was 50° (Figure 2) as compared to the contralateral tibia, which had a TPA of 27°. There was moderate effusion of the left stifle and generalized osteopenia of the left pelvic limb with thinning of the cortices and moderate muscle atrophy. Lameness was attributed to osseous abnormalities, including the ehTPA and, based on the physical examination and radiographic abnormalities, a suspected early partial CCL tear.

Surgery was planned to place a hinged circular external fixator (IMEX Veterinary, Inc., Longview, TX) and perform distraction osteogenesis to sequentially reduce the TPA while simultaneously lengthening the crus (Figure 3). Based on previously described methodology [9, 10], the sagittal plane proximal tibial mechanical axis was established by transferring the normal joint reference angle from the contralateral tibia. The sagittal plane distal tibial mechanical axis was estimated based on the course of the mechanical axis in contralateral tibia, because of the morphologic anomalies of the tibia resultant from the healed diaphyseal fracture. The intersection of these two axes defined the center of rotation of angulation (CORA). Specifically, this was the neutral CORA and correction of the deformity through this point theoretically would neither increase nor decrease the length of the involved limb segment. The transverse bisecting line, which is a line equidistant from each of the mechanical axes that extends through the neutral CORA, was determined. The angular correction axis, which would be defined by placement of the hinge axis at surgery, was positioned on the transverse bisecting line at the apex of the tibial tuberosity. Positioning the hinge axis cranial and remote to the neutral CORA, was done to induce trapezoidal separation of the osteotomy, and thus lengthening of the crus during postoperative distractions [10–12]. Prior to surgery, a graphical trial and error method was used to evaluate the expected results of distraction which projected reducing the TPA and lengthening the crus while inducing minimal new osseous conformational abnormalities.

The dog was anesthetized and positioned in dorsal recumbency. Perioperative cefazolin was administered (22 mg/kg IV q90 minutes, then q8h overnight). Arthroscopy of the left stifle was initially performed and although the stifle was effused and inflamed, there was no cruciate ligament, meniscal, or articular cartilage pathology observed. Fluoroscopy was utilized during the remainder of the procedure to facilitate accurate identification of the proximal tibial physis, positioning of the osteotomies, and placement of fixation elements. The cranial portion of the proximal tibial physis was identified with a hypodermic needle and ablated with electrocautery. A subperiosteal left proximal fibular diaphyseal osteotomy was performed via a lateral approach. The left tibia was accessed via a medial approach and the plate and screws were removed. An osteotomy was initiated at the proximomedial aspect of the tibial diaphysis using a 24 mm TPLO saw blade. The same saw blade was used to perform a second concentric osteotomy to excise 2–3 mm of bone from the lateral margin of the distal tibial segment; this osteotomy was directed obliquely in a distolaterally direction to address the varus deformity. The margins of the tibial segments were opposed and two convergent interfragmentary 1.6 mm Kirschner wires were placed to stabilize the tibial segments. A third 1.6 mm Kirschner wire was placed transversely, from medial-to-lateral, through the apex of the tibial tuberosity: accurate position of this Kirschner wire was confirmed using fluoroscopy. Cannulated hinge assemblies were placed over each of the segments of this Kirschner wire that protruded medial and lateral to the tibial tuberosity. The proximal portion of each hinge assembly was secured directly to a 118 mm stretch ring with the open section of the ring positioned caudal. The stretch ring was oriented parallel with the tibial plateau, and then was affixed to the proximal tibial metaphysis with two opposing 1.6 mm olive wires and two 1.6 mm Kirschner wires. A 150 mm threaded rod was secured in the distal component of each of the hinge assemblies and these rods were secured distally to a complete 118 mm ring positioned circumferentially around the distal crus. The complete ring was secured to the distal tibial diaphysis using a single 1.6 mm Kirschner wire and two opposing 1.6 mm olive wires. Three 3.2 mm partially threaded half-pins, mounted on the distal ring using a hybrid rod or a two-hole post, were placed in the medial and cranial tibial diaphysis. A pair of angular motors were attached caudolaterally and caudomedially, articulating the two rings (Figure 4). Immediate post-operative radiographs confirmed appropriate location of the tibial and fibular osteotomies as well as the hinge axis (Figure 5(a)). The varus deformity had been over-corrected resulting in 12° of tibial valgus angulation with mild persistent internal rotation of the distal tibia.

Distraction was initiated the day following surgery at a rate of 1 mm per day as measured along the caudal cortex of the tibia with a rhythm of three distractions daily. The dog was administered methadone (0.1 mg/kg IV q4h) for 1 day, carprofen (2 mg/kg PO q12h) for 14 days, tramadol (4 mg/kg PO q8h) for 10 days, and cephalexin (40 mg/kg PO q12h) for 28 days. Radiographs were obtained 2 weeks following surgery and there was a 12 mm gap separating the caudal margins of the osteotomy. There was cranioproximal rotation of the proximal tibial segment about the hinge axis with concurrent separation of the fibula and early regenerate bone was present in the distraction gaps. In this and all subsequent radiographs there was pronounced soft tissue swelling, particularly laterally, associated with the proximal fixation wires. The talocrural joint was noted to be maintained in extension in all imaging documenting the distraction process. Distraction was terminated 19 days post-operatively, with 6 mm and 17 mm of distraction along the cranial and caudal tibial cortex, respectively, and continued regenerate bone formation in both distraction gaps (Figure 5(b)).

The dog was placing moderate weight on the left pelvic limb when re-evaluated 4 weeks following surgery. There was substantial wire tract inflammation, more notably laterally, where the wires on the proximal ring traversed through the extensor muscles. Radiographs showed increased mineralization of the regenerate bone in both the tibial and fibular distraction gaps with fibrous interzones clearly evident. At 6 weeks following surgery, the regenerate bone had undergone extensive consolidation and remodeling, but there was still a narrow, well-marginated 1–2 mm fibrous interzone present in the tibia (Figure 5(c)).

The dog's lameness worsened acutely 7 weeks following surgery. When examined, the dog would not place weight on the left pelvic limb and had marked muscle atrophy. Direct pressure applied to the proximal left calcaneus elicited a pain response and there was minor palpable instability. Radiographs revealed an acute, complete, short-oblique, minimally displaced fracture of the mid-body of the left calcaneus. There was persistent osteopenia and thinning of the cortices of all of the bones of the left pelvic limb. The tibial distraction site had circumferential, well-defined smooth osseous proliferation, but a narrow fibrous interzone was still identifiable.

The dog was anesthetized, the external fixator was removed, and the tibial distraction site was palpably stable. Open surgical stabilization of the calcaneal fracture was performed via a lateral approach. The fracture was stabilized with a laterally positioned 7-hole 2.4 mm locking condylar plate (Synthes Vet, West Chester, PA, USA). During screw placement, the bone was noted to lack mineral density, so a 4-hole 2.0 mm locking compression plate (Synthes Vet, West Chester, PA, USA) was placed on plantar surface of the calcaneus.

The dog's weight-bearing on the left pelvic limb improved steadily over the subsequent 2 weeks and radiographs obtained 14 days following calcaneal fracture stabilization showed indistinct fracture margins with mild thickening of the tarsal regional soft tissues and persistent moderate osteopenia of the distal left pelvic limb. Complete healing of the calcaneal fracture was observed radiographically 6 weeks postoperatively. Radiographs of the crus revealed consolidation of the regenerative bone with recurvatum and remodeling of the proximal tibia. The previously noted osteopenia was subjectively improved. During the subsequent 5 weeks, the dog remained intermittently lame with continued soft tissue swelling and sensitivity to direct pressure applied over the plantar surface of the calcaneus. The plantar plate was removed when the dog was spayed, 11 weeks after the calcaneal fracture had been stabilized, because there were concerns that the plate was irritating the flexor tendons and contributing to the dog's lameness.

The dog was re-evaluated 12 months after surgery and had a subtle weight-bearing left pelvic limb lameness which was only discernable while trotting. Force plate analysis was performed and the peak vertical force (% body weight) and peak vertical impulse (% body weight × second) were 66.3 and 8.2 for the left pelvic limb and 77.5 and 10.8 for the right pelvic limb, respectively. The dog positioned the limb in slight abduction when standing. The right stifle flexion angle was 43° compared to the left which was 36°. The extension angles of the right and left stifles were 156° and 159°, respectively. The right tibiotarsal flexion angle was 34° compared to the left which was 60°. The right and left tibiotarsal extension angles were 163° and 164°, respectively. On palpation of the left stifle, 3 mm of cranial drawer with a distinct end point could be elicited, while the right stifle had 1 mm of cranial drawer. Cranial tibial thrust and pain on hyperextension could not be elicited in either stifle. The circumference of the left thigh was 330 mm and the right thigh was 361 mm. Radiographs of the left crus revealed extensive remodeling of the distraction sites and a TPA of 3° (Figure 5(d)). The left femur (206.7 mm) was 11.7 mm shorter than the right femur (218.4 mm). The left tibia (203.3 mm) was 22.7 mm shorter than the right tibia (226.0 mm). The combined left femoral and tibial length measured 34.4 mm shorter than the combined right femoral and tibial length with mild persistent valgus (12°) angulation. Twenty-eight months after application of the fixator, the owner reported that the dog only exhibited a subtle weight-bearing lameness after strenuous activity.

## 3. Discussion

Traumatic premature closure of the caudal aspect of the proximal tibial physis can result in an ehTPA [1–3]. High TPAs can alter stifle mechanics and induce CCL pathology [2, 4, 5]. The dog in this report sustained a Salter-Harris type III fracture which prematurely closed the caudal proximal tibial physis at 18 weeks of age. Continued growth of the cranial portion of the physis resulted in the dog having a TPA of 50° at 24 weeks of age. An early partial tear of the CCL was suspected because of the dog's mild, but persistent lameness, pain response elicited on hyperextension of the left stifle, moderate stifle effusion and ehTPA. Surgery was purposed to decrease the TPA to 7° to proactively address potential CCL pathology. Second look arthroscopy in dogs with competent partial CCL tears have substantiated that TPLO can mitigate the progression of ligamentous pathology and the development of degenerative joint disease [13]. While this dog's CCL appeared normal during arthroscopy, we cannot exclude the potential of early or microscopic pathology.

The dog in this report had a 13% pelvic limb length discrepancy prior to surgery which may have had a nominal impact on limb function. Franczuzki et al. reported that experimental unilateral shortening of skeletally mature dogs' femori by 20% was not associated with an appreciable, subjective lameness [14]. The length discrepancy between pelvic limbs in a 24-week-old dog was, however, likely to increase as the dog continued to grow. Proximal tibial physis growth dynamics have been studied in Labrador Retrievers and are characterized by a steady, nonlinear reduction in longitudinal growth, which eventually ceases at 13 months of age [15].

We ablated the cranial portion of the proximal tibial physis using electrocautery [16, 17] to mitigate the potential of having continued asymmetric proximal tibial growth which could increase the TPA following distraction. Epiphysiodesis was achieved based on the subsequent radiographic appearance of the physis. Proximal tibial epiphysiodesis using a trans-physeal screw has been described to manage skeletally immature dogs with CCL insufficiency [18]. Whether thermal ablation using electrocautery [16, 17], as performed in the dog in this report, is a suitable modality for performing proximal tibial physeal epiphysiodesis remains unclear.

A hinged circular fixator was utilized in this dog so that distraction osteogenesis could be performed, allowing simultaneous crural lengthening and reduction of the TPA [19–22]. Distraction yielded 6 mm and 17 mm of increased tibial length as measured at the cranial and caudal cortices, respectively. Alterative procedures described to address ehTPA such as a cranial closing wedge ostecotomy, a TPLO performed in conjunction with a secondary osteotomy or a co-planar CORA-based leveling osteotomy would have exacerbated the length discrepancy in the already short affected limb [5, 6, 23, 24].

Prior to surgery the dog had a frontal plane deformity of 8° of varus. We addressed the varus angulation by excising a wedge of bone along the lateral margin of the distal tibial segment. Unfortunately, we inadvertently excised too large a wedge as the dog had 12° of valgus angulation following surgery which likely contributed to the dog's abducted stance at the time of the dog's final long-term evaluation. The dog also had internal tibial torsion prior to surgery. We attempted to acutely correct this torsion prior to placing the Kirschner wires which stabilized the osteotomy, but mild residual torsion was noted on the immediate post-operative radiographs. The torsional alignment to the talocural articulation and pes, however, had improved at the time of the final long-term evaluation.

The hinge axis was placed cranial and remote to the osteotomy to achieve trapezoidal separation of secured bone segments during distraction which resulted in limb segment lengthening while effectively reducing the dog's ehTPA [10–12, 22]. During planning, the neutral CORA was defined by transferring the normal sagittal plane joint reference angle obtained on the contralateral tibia to establish the proximal tibial mechanical axis [10, 12]. The position of the distal tibial mechanical axis was based on the location of that axis on the normal contralateral tibia. Dysplastic abnormalities in tibial plateau and morphologic alterations in the healed tibial diaphysis made accurately defining these axes in the left tibia challenging [10, 12]. Placing the hinge axis along the transverse bisecting line was done to prevent the development of a secondary translational deformity during distraction [10, 12]. There was, however, apparent cranial translation of the proximal tibial segment following distraction resulting in mild unanticipated recurvatum. This recurvatum may have been, at least in part, the result of continued growth from the physis of the tibial tuberosity, as the orientation of tibial tuberosity was redirected cranial and distal during distraction.

Use of hinged circular external fixators to perform distraction osteogenesis demands a high level of post-operative care [25]. The dog in this report had substantial wire tract inflammation induced by the wires secured to the proximal ring. Wire tract morbidity is often associated with the use of wires as fixation elements placed in the proximal tibia, as the wires must be placed through the prominent muscle mass laterally [26–28]. A full ring and three fixation wires were used to secure the distal tibial diaphysis. Wire tract morbidity in this location is minimal as there is a limited amount of muscle surrounding the distal tibia [22, 28, 29]. The construct was hybridized by placing intermediate half-pins in the mid-diaphysis of the tibia. This was done to improve construct stability [30] while limiting post-operative morbidity associated with fixation wires traversing the prominent muscle groups located more proximally in caudolateral crus [22, 29, 28].

In humans, calcaneal insufficiency fractures can result from physiological stress applied to bone with deficient elastic resistance [31]. These fractures occur most commonly in patients with disturbances that either directly or indirectly affect bone metabolism leading to osteomalacia and osteoporosis [32]. The calcaneal fracture sustained by the dog in this report was attributed to extreme tension on the plantar aspect of the tarsus which exceeded the strength of the osteopenic calcaneus [33, 34]. Distraction of the proximal crus can result in contracture of the gastrocnemius muscles [35, 36]. In human patients, tension in the gastrocnemius muscles provides the greatest restraint to tibial lengthening and if left unopposed, subluxation of the knee and plantar extension of the ankle can develop [35]. Musculotendinous contracture in people may require tenotomy or capsulotomy to permit lengthening to continue [35, 36]. Contracture limiting range of motion is more problematic when performing proximal limb distractions [35–39]. McCartney reported that distraction rates of up to 3 mm per day were not associated with long-term deleterious soft tissue complications, but transient musculotendinous contracture developed during lengthening of the femur [37]. The young age of the dog reported here, coupled with aggressive post-operative rehabilitation may have contributed to the eventual satisfactory outcome.

At surgery, the proximal temporary interfragmentary Kirschner wire placed to stabilize the tibial osteotomy was noted to be aligned parallel to the tibial plateau on fluoroscopy. The position of this wire was used to align the stretch ring stabilizing the proximal tibial segment. A post-distraction TPA of 7° was targeted based on previous studies which evaluated eliminating cranial tibial subluxation with TPLO [19, 40]. Measuring the TPA on radiographs during the distraction process was not possible because the proximal ring impeded our ability to view the plateau. We had to estimate the TPA based on the position of the proximal ring and consequently our final TPA was 3° rather than 7°.

Despite the complications that developed during the post-operative convalescent period, use of the hinged circular construct allowed for reduction of the TPA from 50° to 3°, while lengthening the crus. Clinical abnormalities suggestive of CCL insufficiency have yet to develop in this dog's left stifle. The dog in this report had a final tibial length discrepancy of 10% with a left pelvic limb length deficit of 8% and had only a nominal lameness at the time of the final long-term evaluation [41]. While the measured force plate parameters were still less in the left pelvic limb than the right pelvic limb, symmetry between the pelvic limbs had improved substantially at the time of the final evaluation, with a 16% increase in the left pelvic limb peak vertical force and 44% increase in left pelvic limb peak vertical impulse. The valgus resultant from acute over-correction of the preoperative tibial varus was only apparent while the dog was standing and was not appreciable while walking. While our approach to this unusual clinical circumstance of a dog with an ehTPA and crural shortening yielded an acceptable long-term limb functional outcome, consideration should be given to alternative approaches: the ehTPA could have been primarily addressed acutely using an applicable tibial osteotomy [5, 6, 23, 24]. Subsequent lengthening of the crus could have been performed, if limb function was unsatisfactory, through distal tibial and fibular osteotomies which potentially would be associated with less morbidity [22, 42].

## Figures and Tables

**Figure 1:**
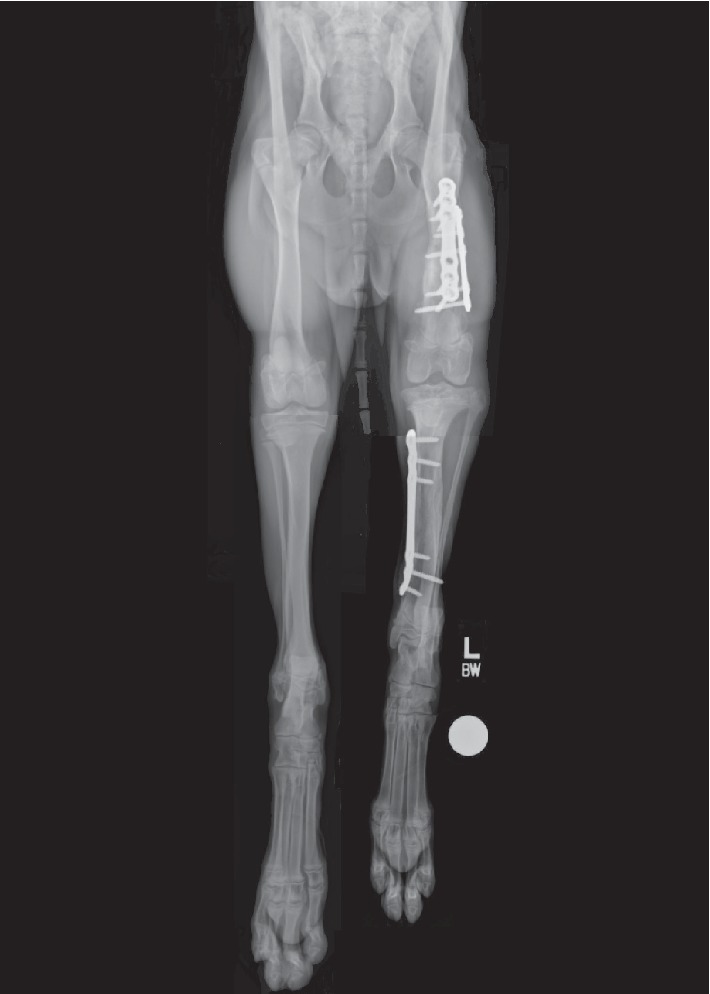
Composite radiograph of the dog's pelvic limbs depicting limb length disparity. These radiographs were obtained 6 weeks following initial fracture stabilization. The left femur was 18% shorter than the right femur. The left tibia was 7% shorter than the right tibia. Left pelvic limb muscle atrophy, proximal tibial varus and internal torsion were also evident.

**Figure 2:**
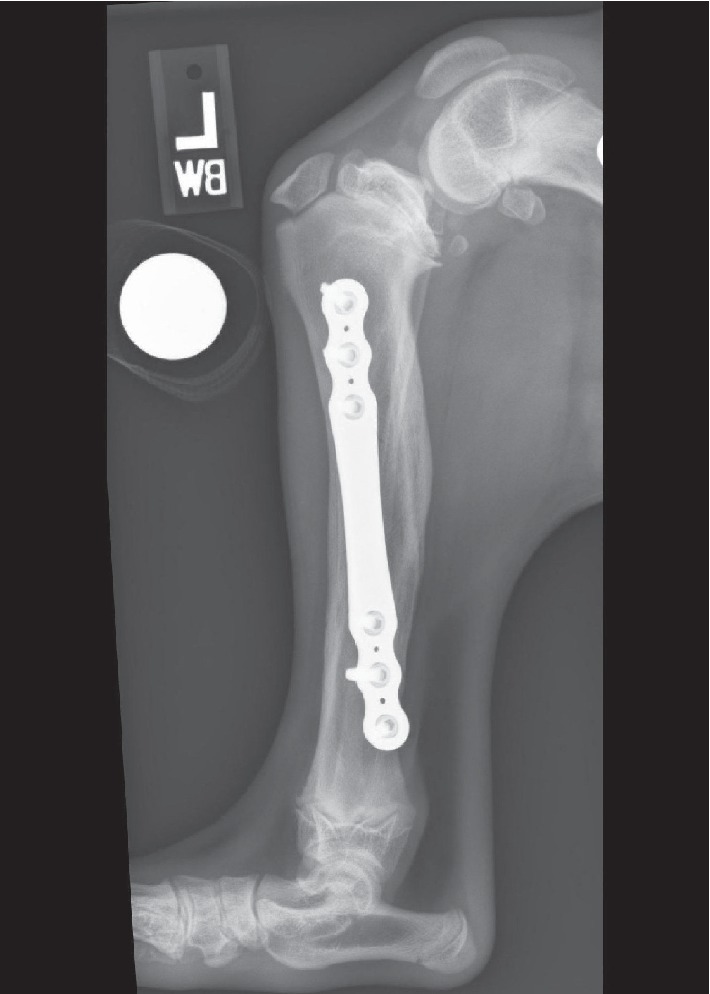
Lateral radiograph of the dog's left pelvic limb obtained 6 weeks following initial fracture stabilization. Premature closure of the caudal portion of the tibial plateau has resulted in a tibial plateau angle of 50°.

**Figure 3:**
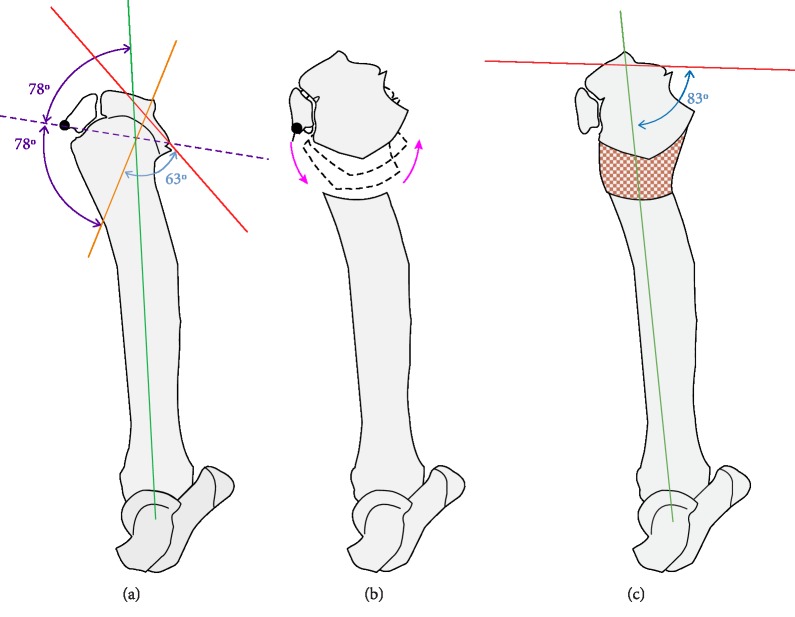
Sagittal deformity correction plan and projected results. (a) The proximal tibial axis (orange line) was established by defining the tibial plateau (red line) and transferring the mechanical caudal proximal tibial angle as measured on the contralateral normal tibia (63°; tibial plateau angle (TPA) of 27°) and positioning that angle to extend proximally through the tibial eminences. The distal tibial mechanical axis (Kelly green line) was estimated based on the position this line extended proximally from the talus on the normal, contralateral limb. The intersection of these two axes defined the neutral center of rotation of angulation (CORA). The transverse bisecting line (purple dashed line), which is a line passing through the neutral CORA and located equidistant (78°) from the proximal and distal tibial axes, was drawn. An opening CORA, located cranially on the transverse bisecting line at the apex of the tibial tuberosity (large black dot), was selected for the location of the hinge axis. (b) A radial osteotomy was planned in the region of the proximal tibial metaphyseal-diaphyseal junction. Distraction would result in rotation of the proximal tibial segment about the hinge axis (magenta arrows) and separation of both the cranial and caudal tibial cortices along the osteotomy margin resulting in lengthening of the limb segment as the TPA was sequentially reduced to 7°. Locating the hinge axis on the transverse bisecting line was done to reduce the TPA and perform lengthening without inducing a secondary translational deformity. During distraction the position of the tibial eminences, which were used as the proximal landmark to define the mechanical tibial axis, were translated slightly cranial which also translated the final mechanical tibial axis (forest green line) cranial. (c) Regenerate bone (orange stippled area) would form in the distraction gap establishing a confluent column of tibial bone.

**Figure 4:**
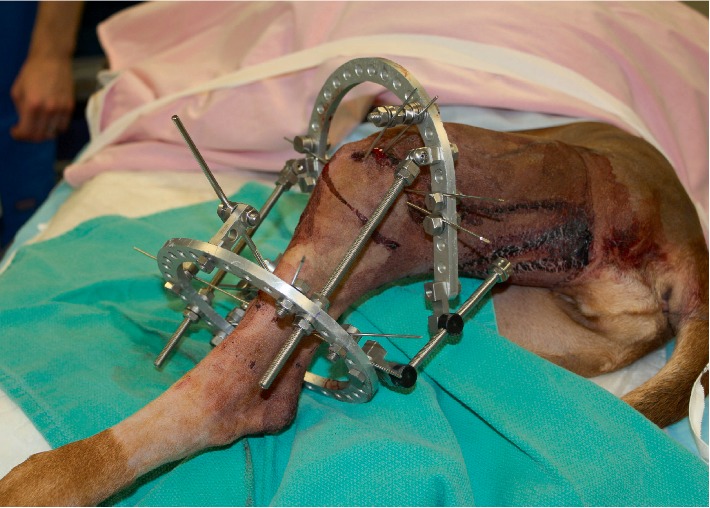
Photograph of the left pelvic limb obtained immediately following surgery. The stretch ring and associated fixation wires stabilized the plateau segment. The hinges, attached directly to the proximal ring, were placed to position the hinge axis through the apex of the tibial tuberosity. One of the two angular motors which were used to perform the distraction is visible caudolaterally. The second, caudomedial motor cannot be seen in this photograph. Half-pins were placed cranial and medial on the distal ring to improve construct stability while minimizing postoperative morbidity.

**Figure 5:**
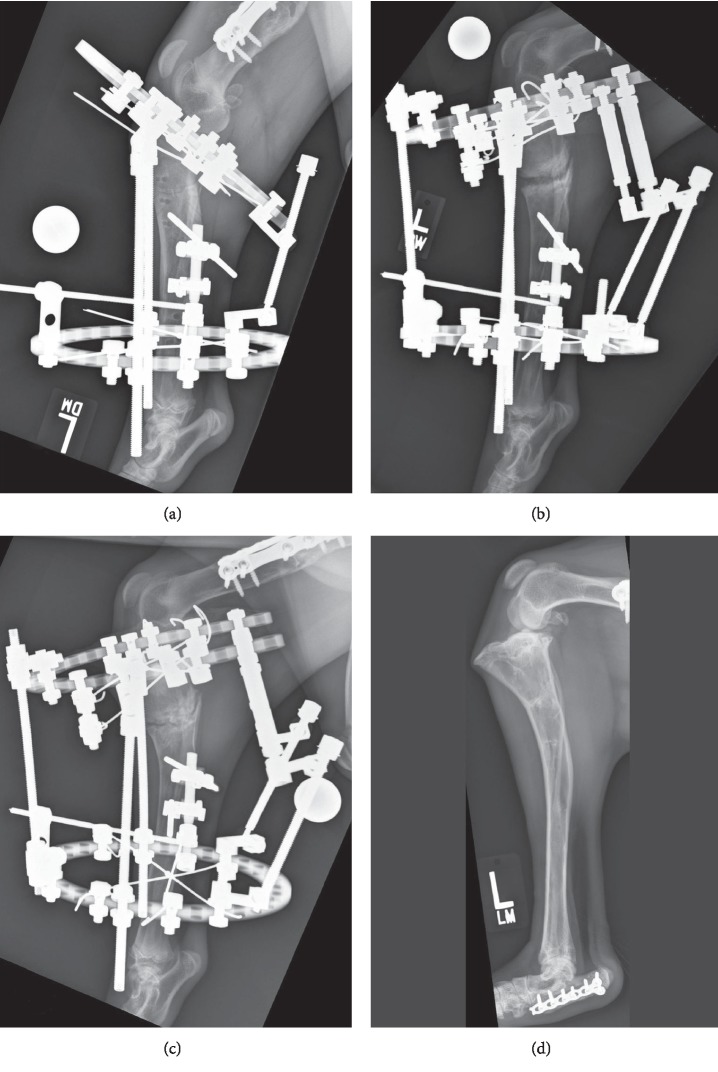
A sequence of lateral postoperative radiographs of the dog's left pelvic limb which were obtained immediately following placement of the fixator (a), 19 days following surgery, at the termination of distraction (b), 6 weeks following surgery (c), and 12 months following surgery at the time of the final long-term follow-up evaluation (d).

## Abbreviations

ehTPA: Excessively high tibial plateau angle
CCL: Cranial cruciate ligament
TPLO: Tibial plateau leveling osteotomy
CORA: Center of rotation of angulation
TPA: Tibial plateau angle.
